# Delayed neutrophil apoptosis enhances NET formation in cystic fibrosis

**DOI:** 10.1136/thoraxjnl-2017-210134

**Published:** 2017-09-15

**Authors:** Robert D Gray, Gareth Hardisty, Kate H Regan, Maeve Smith, Calum T Robb, Rodger Duffin, Annie Mackellar, Jennifer M Felton, Lily Paemka, Brian N McCullagh, Christopher D Lucas, David A Dorward, Edward F McKone, Gordon Cooke, Seamas C Donnelly, Pradeep K Singh, David A Stoltz, Christopher Haslett, Paul B McCray, Moira K B Whyte, Adriano G Rossi, Donald J Davidson

**Affiliations:** 1 UoE/MRC Centre for Inflammation Research, University of Edinburgh, Edinburgh, UK; 2 Carver College of Medicine, University of Iowa, Iowa City, Iowa, USA; 3 Department of Respiratory Medicine, St Vincent’s Hospital, Dublin, Ireland; 4 Department of Medicine, Trinity College Dublin and Tallaght Hospital, Dublin, Ireland; 5 Department of Microbiology, Washington University Medical School, Seattle, Washington, USA

**Keywords:** Cystic Fibrosis, Neutrophil Biology

## Abstract

**Background:**

Cystic fibrosis (CF) lung disease is defined by large numbers of neutrophils and associated damaging products in the airway. Delayed neutrophil apoptosis is described in CF although it is unclear whether this is a primary neutrophil defect or a response to chronic inflammation. Increased levels of neutrophil extracellular traps (NETs) have been measured in CF and we aimed to investigate the causal relationship between these phenomena and their potential to serve as a driver of inflammation. We hypothesised that the delay in apoptosis in CF is a primary defect and preferentially allows CF neutrophils to form NETs, contributing to inflammation.

**Methods:**

Blood neutrophils were isolated from patients with CF, CF pigs and appropriate controls. Neutrophils were also obtained from patients with CF before and after commencing ivacaftor. Apoptosis was assessed by morphology and flow cytometry. NET formation was determined by fluorescent microscopy and DNA release assays. NET interaction with macrophages was examined by measuring cytokine generation with ELISA and qRT-PCR.

**Results:**

CF neutrophils live longer due to decreased apoptosis. This was observed in both cystic fibrosis transmembrane conductance regulator (CFTR) null piglets and patients with CF, and furthermore was reversed by ivacaftor (CFTR potentiator) in patients with gating (G551D) mutations. CF neutrophils formed more NETs and this was reversed by cyclin-dependent kinase inhibitor exposure. NETs provided a proinflammatory stimulus to macrophages, which was enhanced in CF.

**Conclusions:**

CF neutrophils have a prosurvival phenotype that is associated with an absence of CFTR function and allows increased NET production, which can in turn induce inflammation. Augmenting neutrophil apoptosis in CF may allow more appropriate neutrophil disposal, decreasing NET formation and thus inflammation.

**Key messages**

**What is the key question?**

- Do cystic fibrosis (CF) neutrophils live longer and allow the preferential formation of neutrophil extracellular traps (NET)?

**What is the bottom line?**

- CF neutrophils have a primary defect causing decreased spontaneous apoptosis and allowing increased levels of NET formation that can promote inflammation.

**Why read on?**

- This work links a fundamental cystic fibrosis transmembrane conductance regulator-related defect in neutrophil apoptosis to the promotion of inflammation in CF by NETs, and demonstrates that augmenting neutrophil apoptosis may be a therapeutic strategy to reduce NET formation and inflammation in CF.

## Introduction

Cystic fibrosis (CF) is the most common fatal single gene disorder in Caucasian populations with a prevalence of 1 in 2000 live births, and is a multiorgan disease affecting the lungs, pancreas, sweat glands, gut, liver and kidney. The inflammatory response to infection with lower respiratory pathogens in CF is exaggerated with most patients dying from lung disease.^1^ The pathophysiology of CF lung disease is poorly understood but significant factors include impaired bacterial killing, decreased mucus clearance and overexuberant inflammation.^2^ Neutrophils are a key inflammatory cell in the CF lung acting as professional phagocytes, but their interaction with other immune cells in the lung may be as important and requires further investigation.

The ability of neutrophils to undergo spontaneous apoptosis is protective to the host and is central to the resolution of infectious or inflammatory insults.^3^ Several studies have demonstrated defects in apoptosis leading to increased neutrophil survival in CF,^4 5^ but the consequences of this remain unclear. In the absence of spontaneous apoptosis, other mechanisms of neutrophil disposal such as neutrophil extracellular trap (NET) formation (NETosis) may become increasingly important. NETosis has been proposed as an additional mechanism by which neutrophils can kill bacteria, functioning in addition to phagosomal killing.^6 7^ NETosis occurs in stimulated neutrophils (eg, by interleukin (IL)-8, lipopolysaccharide (LPS), phorbol myristate acetate (PMA) or bacteria) which undergo an oxidative burst and emit a network of DNA, histones, antibacterial and potentially proinflammatory proteins. An excess of NETs has been described in the CF airway,^8 9^ which could be due to either increased production or decreased clearance from the CF lung. NETs have also been associated with inflammatory conditions such as arthritis, systemic lupus erythematosus (SLE) and gout,^10–12^ and more recently NETs have been shown to interact with macrophages and prime inflammation in vascular disease.^13^ Therefore, the interaction of NETs with macrophages as a driver of inflammation could be particularly relevant in CF, where macrophages have an overexaggerated response to inflammatory stimuli.^14^

We hypothesised that neutrophil survival is constitutively increased in CF due to decreased apoptosis, allowing more neutrophils to form NETs that will interact with macrophages and promote inflammation.

## Methods

### Collection of samples from patients with CF

Peripheral blood was collected from stable patients with CF attending the Scottish National CF Service. Patients were considered stable if they had not required intravenous antibiotics in the past 2 weeks. Lung transplant patients were excluded. Patients gave written consent and the study was approved by regional ethics committees (East of Scotland Research Ethics Committee, 15/ES/0094, West of Scotland Research Ethics Committee, 11/WS/0074). Anonymous matched healthy controls were recruited locally (Lothian Research Ethics Committee, 08/S1103/38). Sample collection from patients before and after ivacaftor therapy was approved by the St Vincent’s University Hospital, Dublin, Research and Ethics Committee.

### Isolation of human neutrophils and peripheral blood mononuclear cells

Human peripheral blood was collected into 3.8% sodium citrate. Plasma was aspirated following centrifugation of whole blood at 350×*g* for 20 min. Polymorphonuclear cells and peripheral blood mononuclear cells (PBMC) were isolated by 6% dextran sedimentation and separated by discontinuous (72.9, 63.0% and 49.5%) Percoll (GE Healthcare, Buckinghamshire, UK) gradient as described.^15^ Isolated cells were washed twice in cation-free Dulbecco’s phosphate buffered saline (DPBS^-/-^) and then resuspended in appropriate culture media. In some experiments (referred to in the Results section), neutrophils were isolated using Ficoll-Paque (GE Healthcare) dextran sedimentation and hypotonic lysis of residual erythrocytes.

### Cell viability and apoptosis measurement

Isolated neutrophils (5×10^6^/mL) were cultured in 24 or 96-well plates in Iscove’s Modified Dulbecco’s Medium (IMDM) (in some experiments Roswell Park Memorial Medium (RPMI) was substituted) supplemented with 5% autologous serum (in some experiments 10% fetal calf serum (FCS) or no serum was substituted), 1% penicillin and streptomycin, and 1% L-glutamine alone or in the presence of AT7519 (Astex Pharmaceuticals, Cambridge, UK), granulocyte-macrophage colony-stimulating factor (GM-CSF; R&D Systems, Abingdon, UK) or E. coli LPS (Sigma, Dorset, UK) for 24 hours at 37°C, 5% CO_2_. At stated time points, neutrophils were resuspended (1:5) in DPBS^-/-^ supplemented with 25 mM calcium chloride and labelled with Annexin V-FLUOS (Sigma) at 1:500 and 1 µg/mL propidium iodide (PI) before analysis on a BD FACS Scan, FACS Calibur or BD Accuri cell analyzer as described.^16^ Cytocentrifuge preparations were stained with Diff-Quick (Gamidor, Didcot, UK) to assess for morphological changes of apoptosis.

### Western blotting

Western blotting was carried out as previously described,^17^ with the following antibodies: Mcl-1 (1:1000; Santa Cruz, Dallas, TX, USA), BAX (1:1000; Santa Cruz), β-actin (1:50 000; Sigma) and horseradish peroxidase-conjugated secondary antibodies (1:2500; Dako, Cambridgeshire, UK).

### Microscopic detection of NETs

Neutrophils were seeded (5×10^4^/well) into 24-well plates in RPMI with 5% FCS, allowed to adhere for 30 min and then stimulated with 10 nM PMA and incubated for 4 hours at 37°C, 5% CO_2_. In some experiments, they were allowed to adhere for 6 hours in the presence of media alone or media with 1 µM AT7519 and/or 2.5 ng/mL GM-CSF prior to stimulation and further incubation for 4 hours. After the incubation time, 0.15 µM SYTOX green (Invitrogen, Thermo Scientific, UK) was added before bright field and fluorescent (470/22 nm light emitting diodes excitation) images were captured on an EVOS FL cell imaging system. NET formation was quantified as percentage of SYTOX positive NETs per 10× field (NETs % of total cell count on bright field), as described.^15 18^ All samples were plated in duplicate and multiple fields were counted per well.

### NET DNA release kinetic assay

This was based on a published assay.^15 19^ Isolated neutrophils were seeded (5×10^4^/well) in RPMI 1640 media supplemented with 5% FCS into a flat-bottom 96-well plate and allowed to adhere for 30 min at 37°C, 5% CO_2_. NETs were induced by addition of 10 nM PMA and detected at 30 min intervals in a Synergy HT BioTek plate reader by addition of 0.15 µM SYTOX green, a cell permeable nucleic acid stain with excitation/emission spectra of 504/523 nm. In some experiments, 1 µM AT7519 and/or 25 ng/mL GM-CSF were added to the culture media for 6 hours prior to PMA stimulation.

### Human macrophage culture

Isolated PBMCs were seeded in IMDM into 6-well plates for 60 min (Nunc Upcell, ThermoFisher, Waltham, MA). Media was then removed, cells were washed twice in IMDM and remaining adherent monocytes were cultured in IMDM supplemented with 10% autologous serum, 1% penicillin and streptomycin, and 1% L-glutamine for 5 days at 37°C, 5% CO_2_. On day 5, cells were washed twice in DPBS^-/-^ before detachment. Monocyte-derived macrophages (MDM) were then seeded into 48-well tissue culture plates at 2.5×10^5^/well into IMDM supplemented with 10% autologous serum, 1% penicillin and streptomycin, and 1% L-glutamine for a further 1–2 days at 37°C, 5% CO_2_.

### Human NETs and macrophage coculture

Isolated neutrophils were stimulated with 100 nM PMA or dimethyl sulfoxide vehicle control in Hanks' balanced salt solution (HBSS^-/-^) for 15 min in rolling suspension at room temperature (to stop clumping). Neutrophils were then washed three times in HBSS^-/-^ to remove any residual PMA before seeding 5×10^5^/well onto MDMs (2:1 neutrophil to MDM ratio) in 500 µL IMDM supplemented with 10% macrophage-donor serum, 1% penicillin and streptomycin, and 1% L-glutamine for 24 hours at 37°C, 5% CO_2_. PMA-treated neutrophils were considered NETing neutrophils (or NETs) and non-PMA-treated neutrophils as control neutrophils. Supernatants were collected at 24 hours, centrifuged at 300×*g* for 5 min to remove cell debris and frozen at −80°C. RNA was extracted from remaining adherent MDMs using the Direct-Zol RNA extraction kit per manufacturer’s instructions (Zymo Research, Irvine, CA).

### Coculture supernatant ELISA

IL-8 and tumour necrosis factor (TNF) were measured in culture supernatant using commercially available ELISAs (R&D Systems) as per the manufacturer’s instructions.

qRT-PCR of macrophage gene qRT-PCR was performed using commercially available TaqMan gene expression assays (ThermoFisher) for IL8, TNF, CXCL9, CCL17 and 18s RNA as per manufacturer’s instructions. Data were expressed as fold change from control.

### Scanning electron microscopy of bronchial tissue

Bronchial tissue was obtained from an explanted CF lung (under institutional board approval, University of Iowa) and fixed with 2.5% glutaraldehyde in 0.1 M cacodylate buffer and processed for electron microscopy (EM). For full methods, see online supplementary data.

10.1136/thoraxjnl-2017-210134.supp1 Supplementary data

### Statistical analysis

All data are expressed as mean±SE error of the mean. Flow cytometry data were analysed using FlowJo software (TreeStar, Ashland, Oregon) or BD Accuri platform specific software. Data were analysed with GraphPad Prism (La Jolla, CA) by t-test, Mann-Whitey U test or analysis of variance with appropriate post-test as noted in figures.

## Results

### CF neutrophils have increased survival due to less apoptosis

**Figure 1:**
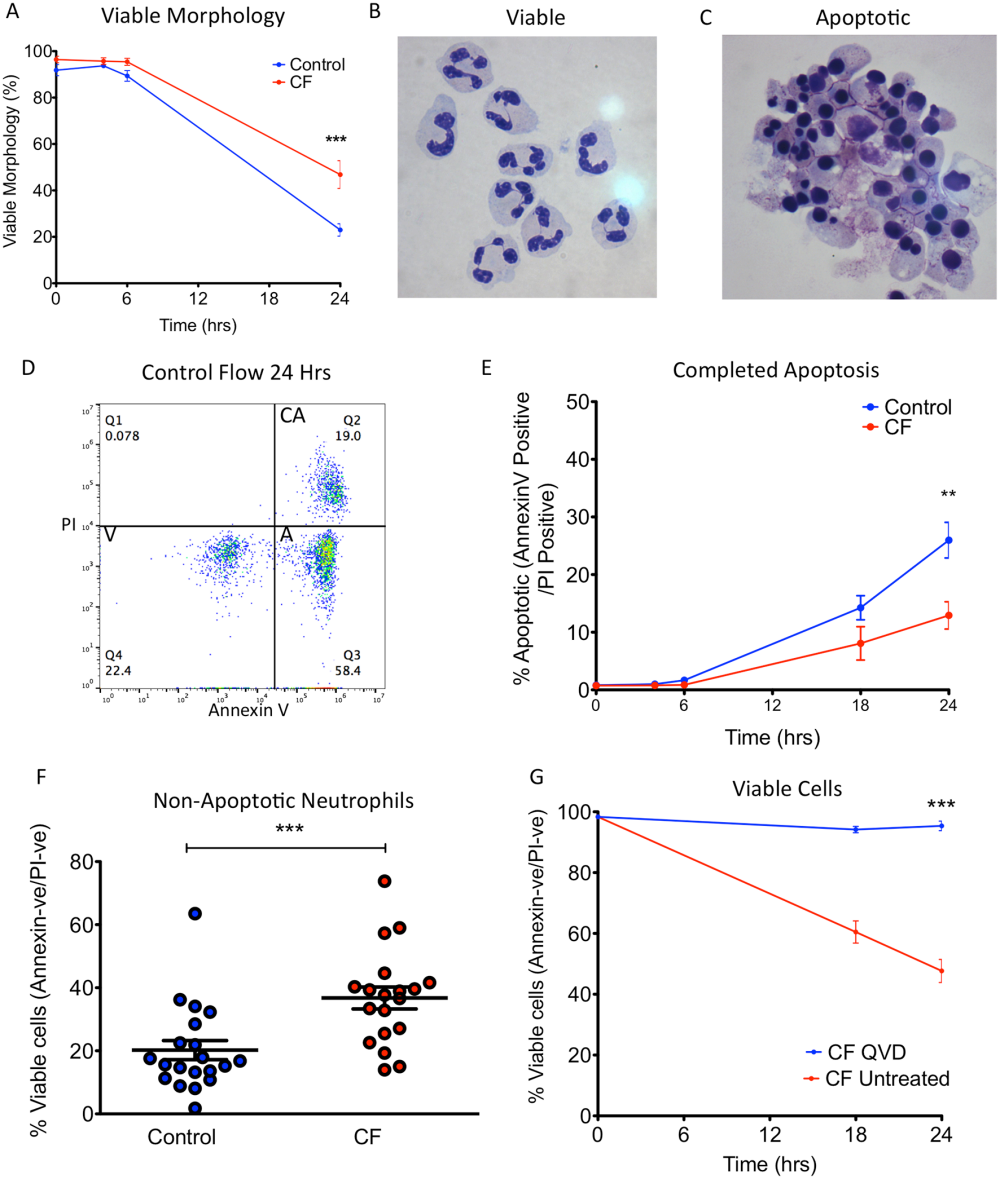
CF neutrophils have increased survival due to less apoptosis. (A) Cultured CF neutrophils are more viable at 24 hours than healthy controls when assessed by morphology (n=6 CF, 5 controls). (B) Freshly isolated viable neutrophils in ex vivo culture demonstrating multilobed nuclei (×100 objective). (C) Apoptotic neutrophils following prolonged ex vivo culture demonstrating characteristic nuclear condensation (×100 objective). (D) Flow plot of control neutrophils at 24 hours ex vivo culture demonstrating small numbers of viable cells (V=annexin V−ve/PI−ve cells) with the majority of cells having entered apoptosis (A=annexin V+ve/PI−ve) or completed apoptosis (CA=annexin V+ve/PI+ve). (E) Control neutrophils have a higher rate of completed apoptosis than CF (n=19 CF and 20 controls, for patient details see online supplementary table E1). (F) More viable neutrophils are present in CF culture at 24 hours (n=19 CF and 20 controls). (G) Addition of the pan-caspase inhibitor Q-VD-OPh hydrate to CF neutrophil culture increases survival by inhibiting constitutive apoptosis. For patient details, see online supplementary table E1. Data presented as mean±SEM. Analysis with two-way analysis of variance and Bonferroni (A, E, G), unpaired t-test (F). **p<0.01; ***p<0.001. CF, cystic fibrosis; PI, propidium iodide; QVD, Q-VD-OPh hydrate.

CF neutrophils displayed increased survival when assessed by morphology on cytocentrifuge preparations (figure 1A–C, p<0.001). We confirmed this using flow cytometry by Annexin V and PI staining. Control neutrophils displayed more completed apoptosis at 24 hours than CF neutrophils, (figure 1D,E, p<0.01), and at 24 hours more neutrophils were viable in the CF samples suggesting a prosurvival phenotype secondary to a decrease in apoptosis (figure 1F, p=0.0004). Pan-caspase inhibition effectively inhibited all cell death in CF neutrophils (figure 1G, p<0.001) confirming that apoptosis is the major mechanism of neutrophil death in CF but is delayed in comparison to healthy controls.

### Delayed neutrophil apoptosis in CF is related to a loss of cystic fibrosis transmembrane conductance regulator function

**Figure 2:**
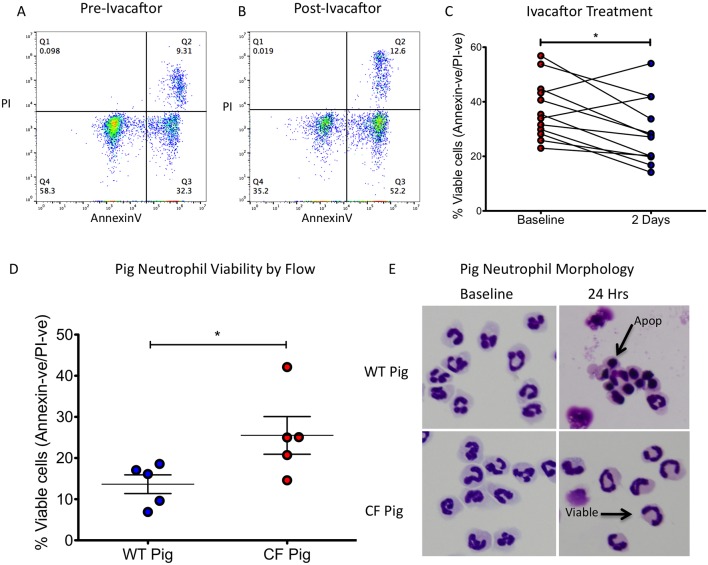
Delayed neutrophil apoptosis in CF is related to a loss of cystic fibrosis transmembrane conductance regulator (CFTR) function. Neutrophils were harvested from 12 patients with at least one G551D mutation before and after starting the CFTR potentiator ivacaftor (for patient details, see online supplementary table E2), and cultured ex vivo for 24 hours. (A) and (B) demonstrate example flow plots (following 24 hours ex vivo culture) from an individual patient before (A) and 2 days after (B) starting ivacaftor, showing that neutrophil survival decreased with treatment. (C) Combined data for 12 patients receiving ivacaftor, neutrophil viability decreased significantly with treatment. (D) Neutrophils were harvested from 2-week-old CF piglets and wild-type (WT) controls. Flow cytometry following 24 hours ex vivo culture demonstrated increased survival in CF (5 CF pigs and 5 WT controls). (E) Representative cytocentrifuge preparations of CF and WT neutrophils at 0 and 24 hours (×100 objective) demonstrating increased numbers of apoptotic neutrophils in WTs at 24 hours. Data presented as mean±SEM. Analysis with paired t-test (C), Mann-Whitney U test (D). *p<0.05. CF, cystic fibrosis; PI, propidium iodide.

Next we assessed whether the delayed apoptosis in CF was related to decreased cystic fibrosis transmembrane conductance regulator (CFTR) function in neutrophils. We obtained neutrophils (using Ficoll-Paque and red cell lysis) from 12 patients with at least one G551D mutation prior to and 2 days after commencing ivacaftor therapy (150 mg twice daily), a CFTR potentiator drug which increases conductance of abnormal CFTR and has a proven clinical effect.^20^ The samples were obtained as part of a previously published observational study,^21 22^ but prepared and analysed separately of the other work. Neutrophil survival at 24 hours was significantly decreased after ivacaftor therapy, suggesting that CFTR potentiation reverses the prosurvival phenotype in CF neutrophils (figure 2A–C, p=0.014). This effect was also seen when patients had treatment for 7 days (data available for six patients, online supplementary figure S1, p=0.0313). To ensure that the apoptosis inducing effects of ivacaftor were not a non-specific effect of the drug, we tested the ability of Ivacaftor to induce neutrophil apoptosis in healthy control neutrophils in culture. No effect was demonstrated after 20 hours of in vitro culture over a range of doses (online supplementary figure S2).

10.1136/thoraxjnl-2017-210134.supp2 Supplementary figure s1

10.1136/thoraxjnl-2017-210134.supp3 Supplementary figure s2

Next we investigated whether the neutrophil survival phenotype was related to the absence of CFTR in neutrophils by utilising CFTR null neutrophils from CFTR^-/-^ piglets (CF piglets). Neutrophils were harvested (using Ficoll-Paque and red cell lysis) from 2-week-old CF piglets and wild-type controls and cultured ex vivo in the presence of 10% FCS. CF piglet neutrophils demonstrated prolonged survival, suggesting that increased neutrophil survival (due to decreased apoptosis) is a primary defect related to absence of CFTR from neutrophils (figure 2D,E, p=0.027).

### Prolonged CF neutrophil lifespan is not caused by inflammation

**Figure 3:**
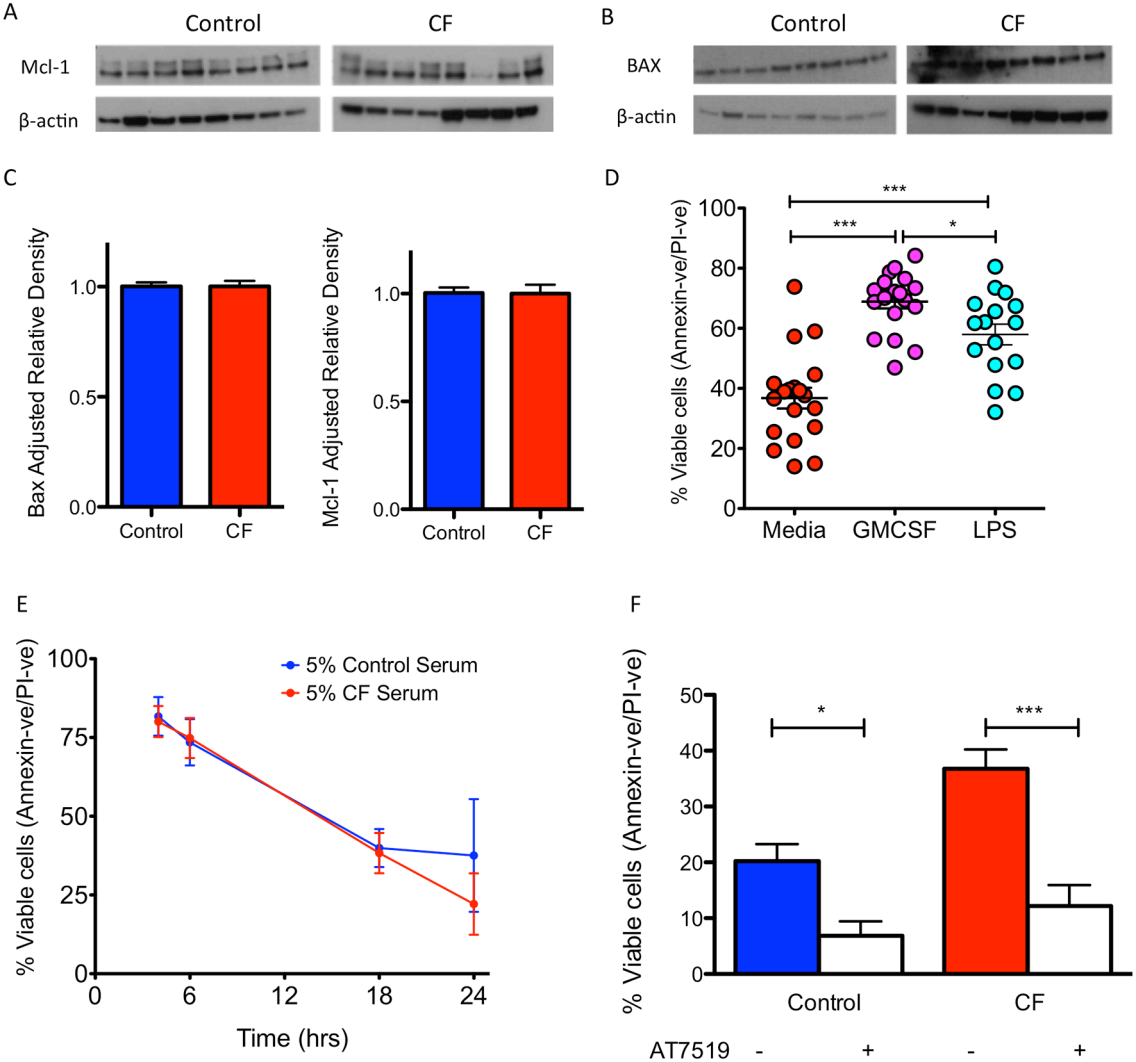
Prolonged CF neutrophil lifespan is not caused by inflammation. CF neutrophils have normal baseline apoptotic signalling of (A) Mcl-1, a 37 kDa antiapoptotic protein, and (B) BAX, a 21 kDa proapoptotic protein when freshly isolated (n=8 CF and 8 healthy controls, for patient details see online supplementary table E3). (C) Mcl-1 and BAX densitometry. (D) CF neutrophils retain sensitivity to delayed apoptosis with prosurvival stimuli GM-CSF (20 ng/mL) and LPS (10 ng/mL) for 24 hours (n=19 CF and 20 controls, for patient details see online supplementary table E1). (E) The primary apoptosis defect in CF is not due to a circulating CF serum factor. Healthy control neutrophils were cultured in media containing 5% pooled CF serum and this did not lead to increased neutrophil survival (n=3 separate healthy donors). (F) The survival defect in CF could be corrected by culture with AT7519 (1 µM) for 24 hours to augment neutrophil apoptosis and effectively reduce survival to healthy control levels (n=19 CF and 20 controls, for patient details see online supplementary table E1). Data presented as mean±SEM. Analysis with unpaired t-test (C), one-way analysis of variance with Newman-Keuls post-test (D, F). *p<0.05; **p<0.01; ***p<0.001. CF, cystic fibrosis; GMCSF, granulocyte-macrophage colony-stimulating factor; LPS, lipopolysaccharides; PI, propidium iodide.

Inflammation has been suggested to prolong neutrophil lifespan by inducing changes in the expression of Bcl-2 family members, in particular Mcl-1^23^ and BAX.^5^ Using samples from patients with CF, we demonstrated no difference in the expression of Mcl-1 and BAX between CF neutrophils and healthy controls (figure 3A–C), suggesting that inflammation-induced dysregulation of intrinsic apoptosis-regulating proteins was not the cause of the prolonged survival of CF neutrophils. Furthermore, the addition of prosurvival stimuli GM-CSF or LPS to CF neutrophils augmented the prosurvival phenotype, demonstrating that activating factors could further enhance the increased neutrophil survival in CF (figure 3D, p<0.001), rather than being the underlying mechanism. To assess whether a serum factor related to the inflammatory environment did not cause the apoptosis delay, we cultured healthy control neutrophils in the presence of CF serum. The addition of 5% pooled CF serum to healthy neutrophil cultures did not increase neutrophil survival (figure 3E). Next we asked whether we could therapeutically reverse the apoptosis delay in CF neutrophils. Culture of CF neutrophils in the presence of AT5719, a cyclin-dependent kinase (CDK) inhibitor (CDKi) known to induce neutrophil apoptosis,^17^ significantly induced apoptosis in control (p<0.01), and CF neutrophils (p<0.001), effectively correcting the apoptosis delay in CF to that of control levels (figure 3F).

### CF neutrophils form more NETs than healthy controls due to their prosurvival phenotype

**Figure 4:**
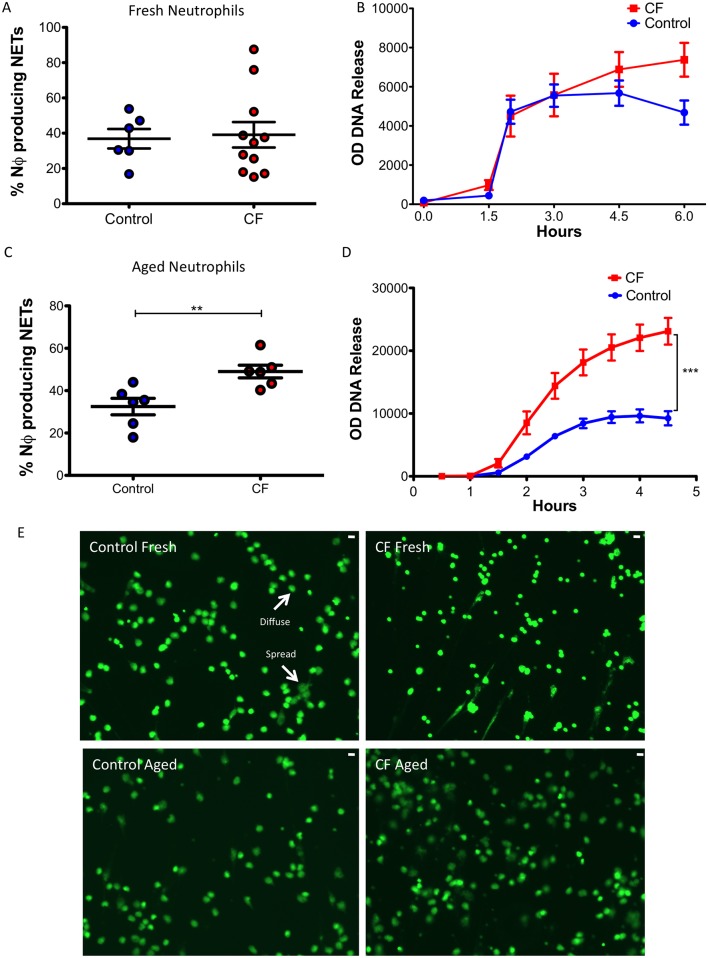
CF neutrophils form more NETs than healthy controls under conditions of ageing in culture. (A) Freshly isolated CF neutrophils form similar amounts of NETs as healthy controls (11 CF vs 6 controls, for patient details see online supplementary table E3) following stimulation with 10 nM phorbol myristate acetate (PMA). (B) DNA release as surrogate marker of NET production yielded similar results from PMA-treated freshly isolated neutrophils (7 CF vs 6 controls), although we observed a non-statistically significant increase in DNA release at later time points by CF neutrophils. (C) Neutrophils aged in culture for 6 hours prior to stimulation with PMA (10 nM) demonstrated increased NET production by CF neutrophils versus controls (n=6 CF vs 6 controls). (D) Increased NET production in aged neutrophils was confirmed by DNA release assay (n=6 CF vs 6 controls). (E) Representative fluorescent microscopy following addition of SYTOX green in non-fixed cells, demonstrating increased PMA-induced NETs in CF following neutrophil ageing compared with controls (and also non-aged CF). Both diffuse morphology and spread morphology NETs are seen (scale bar=10 µm). Data presented as mean±SEM. Analysis with unpaired t-test (A, C), two-way analysis of variance with Bonferroni (D). **p<0.01; ***p<0.001. CF, cystic fibrosis; NET, neutrophil extracellular trap; OD, optical density.

**Figure 5:**
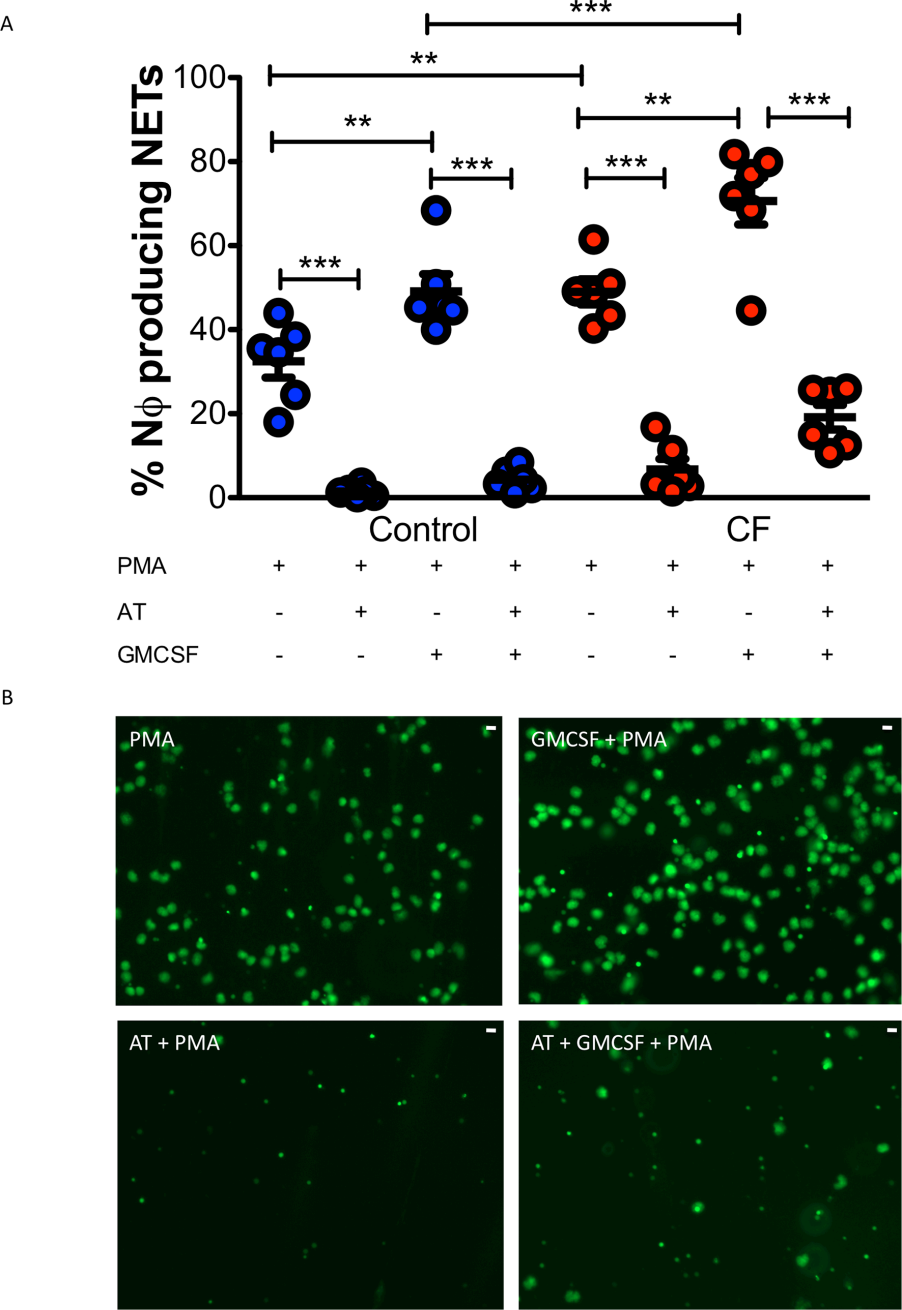
CF neutrophils form more NETs due to their prosurvival phenotype. (A) Healthy control and CF neutrophils were cultured for 6 hours in the presence of GM-CSF (2.5 ng/mL)±AT7519 (1 µM) and then stimulated with PMA (10 nM). The addition of GM-CSF to control neutrophils increased NET formation to that of CF levels. This effect was reversed by AT7519, suggesting that inducing apoptosis stopped NET formation (n=6 CF and 6 healthy controls). (B) Representative fluorescent microscopic images showing SYTOX positive diffuse NETs in culture, enhanced by GM-CSF and inhibited by AT7519 in a patient with CF (scale bar=10 µm). Data presented as mean±SEM. Analysis with one-way analysis of variance with Newman-Keuls post-test (A). **p<0.01; ***p<0.001. CF, cystic fibrosis; GMCSF, granulocyte-macrophage colony-stimulating factor; NET, neutrophil extracellular trap; PMA, phorbol myristate acetate; AT, AT7519.

As CF neutrophils were characterised by reduced apoptosis, we then asked whether they were consequently more susceptible to NETosis. Freshly isolated human CF neutrophils formed NETs as efficiently as healthy control neutrophils in response to 10 nm PMA (figure 4A) after 4 hours of culture. This was confirmed by assessing NET formation by DNA release assay (figure 4B,E). However, DNA release from CF neutrophils diverged from control neutrophil DNA release at 6 hours, suggesting that CF neutrophils formed more NETs than non-CF controls as they aged in culture, and failed to engage in apoptosis (figure 4B). We therefore assessed whether aged CF neutrophils could form more NETs than aged non-CF control neutrophils. Neutrophils were aged for 6 hours and then stimulated with 10 nM PMA to induce NETs. CF neutrophils formed significantly more NETs than similarly aged non-CF control neutrophils (figure 4C, p=0.0087, and figure 4D, p<0.001). We then assessed whether either adding prosurvival factors to control neutrophils would enhance NET formation, or inducing early apoptosis in CF neutrophils would reduce NET formation. The addition of the GM-CSF to non-CF control neutrophils for 6 hours prior to PMA stimulation increased NET formation (figure 5A, p<0.01). Conversely, NET formation in CF neutrophils was inhibited by CDKi (figure 5A,B, p<0.001).

### NETs stimulate an inflammatory response from macrophages and this is exaggerated in CF

**Figure 6:**
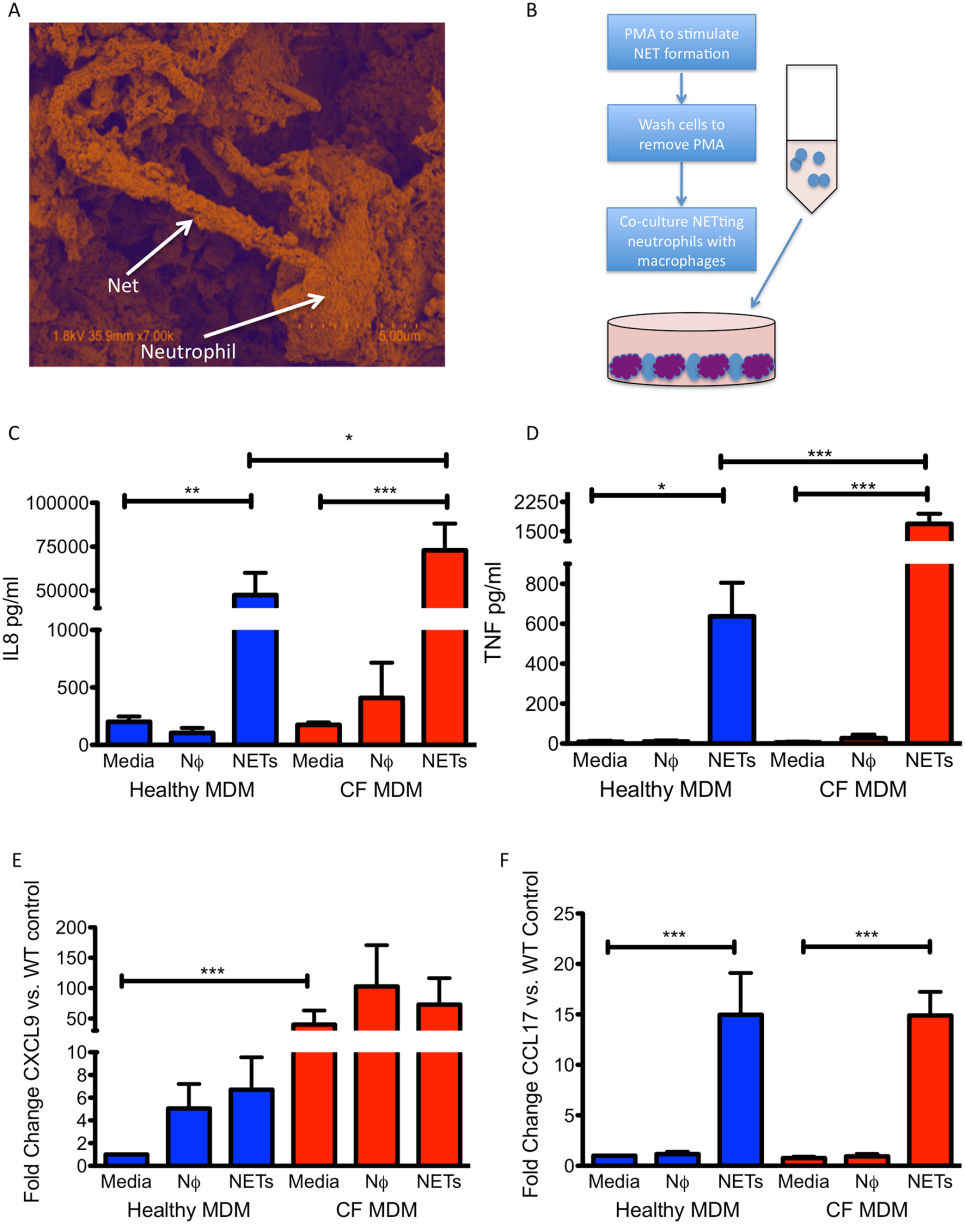
NETs stimulate an inflammatory response from macrophages and this is exaggerated in CF. (A) Scanning electron microscopy of bronchus from patient with explanted CF lung showing characteristic strands of NET-like material associated with neutrophils and bacteria. (B) Outline of novel NET/MDM coculture system. (C) NETs are proinflammatory to MDMs, causing an increase in IL-8 production after 24 hours of coculture and this effect is more pronounced with CF MDMs (n=8 CF and 7 healthy MDM donors, for patient details see online supplementary table E3). (D) NETs induce TNF production from MDMs. (E) NETs induce CCL17 expression in healthy and CF MDMs. (F) NETs induce a non-significant increase in CXCL-9 expression in healthy controls, whereas CXCL-9 is overexpressed at baseline in CF. Data presented as mean±SEM. Analysis by one-way analysis of variance with Newman-Keuls post-test (C–F). **p<0.01; ***p<0.001. CCL, chemokine (C-C motif) ligand; CF, cystic fibrosis; CXCL, chemokine (C-X-C motif) ligand; IL, interleukin; MDM, monocyte-derived macrophage; NET, neutrophil extracellular trap; PMA, phorbol myristate acetate; TNF, tumour necrosis factor; WT, wild type.

We used scanning EM to demonstrate the presence of NETs in the lungs of a patient with CF undergoing lung transplantation (figure 6A) based on morphology and features similar to previous descriptions of NETs on EM.^9^ Next we developed a technique to enable the coculture of NETs (NETing neutrophils) and MDMs at consistent ratios by stimulating neutrophils with 100 nM PMA under rolling conditions, washing and then adding them to MDMs where they then form NETs demonstrating classic morphology after 4–6 hours (online supplementary figure S3). Supernatants and RNA were harvested from cocultures at 24 hours (figure 6B). The addition of NETs to healthy volunteer MDMs induced IL-8 and TNF production (figure 6C,D, p<0.01 and p<0.05, respectively). This effect was also observed when NETs were added to CF MDMs (figure 6C,D, p<0.001 both). The IL-8 and TNF response to NETs was exaggerated in CF MDMs compared with healthy volunteers (figure 6C,D, p<0.05 and p<0.001, respectively). qRT-PCR confirmed increased IL-8 and TNF expression (not shown), and demonstrated that NETs induced the expression of CCL17, a marker of alternative activation in macrophages,^24^ in both CF and control MDMs (figure 6E, p<0.001). Of note, CF MDMs had increased basal expression of the classical macrophage activation marker CXCL9 (figure 6F, p<0.001),^25^ which was further augmented by NETs (although without statistical significance). Overall, these data suggest that CF MDMs interacting with NETs have phenotypic markers of both classical and alternative activation.

10.1136/thoraxjnl-2017-210134.supp4 Supplementary figure s3

## Discussion

Our data demonstrate that CF neutrophils survive longer due to delayed apoptosis and this is associated with the absence of CFTR function. Furthermore, this delay in apoptosis allows CF neutrophils to form NETs more efficiently than healthy control neutrophils, which may be an important proinflammatory mechanism. Impairment of neutrophil apoptosis in CF has been reported,^4 5 26^ and previous studies have suggested that this is either a response to systemic inflammation^5^ or, alternatively, a specific defect in CF neutrophils.^4 26^ The reversal of the apoptosis delay in patients with CF treated with (the CFTR potentiator) ivacaftor suggests that CFTR function in neutrophils is associated with apoptosis pathways. Indeed, previous studies have demonstrated direct effects of CFTR potentiation with ivacaftor on neutrophils,^27 28^ suggesting a fundamental role for CFTR in a number of neutrophil functions, consistent with CFTR being expressed at a biologically significant level.^29^ Our observation that apoptosis is impaired in neutrophils from CFTR null piglets underlines the potential primary nature of delayed neutrophil apoptosis in CF. CFTR has been demonstrated to have a functional role in phagolysosomal activity in both macrophages^30^ and neutrophils,^31 32^ and the absence of CFTR promotes inflammatory cytokine release from macrophages.^14 33^ CFTR has also been implicated in the regulation of T cell suppression by myeloid suppressor cells^34^ and, as such, a diverse but functional role of CFTR in myeloid cells has now been established.

Our data demonstrate that the prolonged survival of CF neutrophils cannot entirely be explained by inflammation in CF. The Bcl-2 family pro and antiapoptotic proteins BAX and McL-1 have been implicated in inflammation-induced neutrophil survival. Mcl-1 excess is observed in neutrophils from patients with sepsis leading to prolonged neutrophil survival,^23^ but we demonstrated no difference in expression between CF and healthy control neutrophils. Inflammation-induced BAX deficiency has been suggested as a cause of delayed neutrophil apoptosis in CF and pneumonia,^5^ but again there was no difference in expression between CF and healthy control neutrophils. Our data infer that the apoptosis delay in CF is a primary feature of CF neutrophils and not simply related to inflammation, further emphasised by CF serum failing to prolong survival in healthy control neutrophils. Exposure of CF neutrophils to prosurvival stimuli such as GM-CSF or LPS further enhances survival, which may be particularly relevant once neutrophils have reached the inflammatory environment of the CF lung. The CF lung environment has been shown to induce changes in neutrophil behaviour and phenotype,^35 36^ and is therefore likely to accentuate the prosurvival phenotype that we demonstrate in CF.

We restored CF neutrophil apoptosis to healthy control levels by using the CDKi AT7519, a strategy that has been demonstrated to decrease inflammation in sterile and infective models of inflammation.^17 37^ Apoptosis is a well-described route of disposal for potentially toxic and damaging neutrophils and the phagocytosis of these apoptotic cells drives inflammation resolution.^3^ Targeting neutrophil apoptosis as an anti-inflammatory strategy in CF is therefore attractive, as it would offer a universal therapy not dependent on a patient’s individual CF genotype. Furthermore, engaging CF neutrophils in apoptosis more effectively may avoid more toxic forms of cell disposal such as NETosis.

We demonstrate for the first time that CF neutrophils form more NETs than healthy controls. Several lines of evidence highlight the importance of NETosis in CF. Excess amounts of DNA in the CF airway have been regarded as a potential contributor to lung disease since the 1960s, although the presence of DNA in the form of NETs is a more recent finding.^8 9 38 39^ These studies, however, fail to definitively address whether CF neutrophils form more NETs than healthy control cells. Our data demonstrate that CF neutrophils form more NETs than controls and this is directly related to the reduction in CF neutrophil apoptosis. The evidence for this is severalfold. First, the major difference in NET formation is observed when neutrophils are aged prior to PMA stimulation by which time control neutrophils, although viable, will have engaged early apoptotic machinery. Second, if non-CF neutrophils are cultured in the presence of GM-CSF before PMA stimulation (to delay apoptosis), they form equivalent numbers of NETs to CF neutrophils. And finally, culture of CF neutrophils for 6 hours in the presence of CDKi before PMA stimulation reduces the level of NET formation in CF to that of healthy controls. Taken together, these data suggest that CF neutrophils form more NETs because they are less able to engage in the normal process of apoptosis, but this can be reversed by augmenting apoptosis with CDKi, in keeping with other studies suggesting that the engagement of several non-apoptotic pathways is required for NET formation.^40–42^ These data also suggest that CDKi may offer a viable therapeutic strategy to subvert NETosis in CF and drive cells towards apoptosis and thus promote inflammation resolution. CDKs are present in a number of isoforms and have a diverse role in cell behaviour, although the major isoforms involved in the regulation of neutrophil apoptosis are CDKs 7 and 9.^43 44^ CDKi may also affect other kinases, for example, glycogen synthase kinase 3 (GSK-3), and in particular the GSK-3β isoform.^45^ Although it is worth noting that GSK-3α is the major isoform found in neutrophils,^46^ this off-target effect may be less important in the context of the present work. Further study of the role of CDKs 7 and 9 in CF neutrophil apoptosis and NETosis will therefore be essential in our understanding of these processes.

There has been recent acknowledgement that granulocyte populations may display a degree of phenotypic heterogeneity leading to the description of low-density neutrophils (LDNs).^47^ Interestingly, a subclass of LDNs, low-density granulocytes (LDGs) may form greater numbers of NETs than ‘normal’ granulocytes in SLE,^48^ although the opposite findings have been described in the study of NETosis in rheumatoid arthritis.^49^ We did not investigate the role of LDNs in the present study, but the contribution of LDNs/LDGs to NET formation in CF would merit further investigation going forward.

We demonstrate that NETs are a potent proinflammatory signal to macrophages. The ability of NETs to kill bacteria has been described since their first description in 2004,^6^ but the potential inflammatory consequences of the presence of NETs in CF have not. We developed an assay to assess whether NETing neutrophils cultured at a consistent ratio with macrophages could promote the release of proinflammatory cytokines (from macrophages), and whether CF macrophages were more susceptible to this stimulus. NETs induced IL-8 and TNF release from MDMs in contrast to previous data.^50^ Our data are however consistent with the recent finding that NETs can prime macrophages for further cytokine release.^13^ We demonstrated an enhanced cytokine release from CF MDMs in response to NETs, consistent with CF macrophages being hyper-responsive to proinflammatory stimuli.^14^ We also demonstrate that the interaction of NETs with CF MDMs is associated with the expression of both classical and alternative markers of macrophage activation and would speculate that this could be a contributing factor to the non-resolving (frustrated) inflammation in CF. Although not addressed in this study, CF epithelial cells are a central part of the innate immune system in CF and as such the interaction of NETs with epithelial cells is of significant interest and will be subject to future study.

We acknowledge a number of limitations in this present study. First, we used peripheral blood neutrophils to study apoptosis and NETosis in the context of CF. Airway neutrophils may have a distinct phenotype in CF,^35 36^ and as such further study of the processes described in this paper (in airway neutrophils) may be indicated. Nevertheless, peripheral neutrophils have been used previously to assess neutrophil function,^4 27 29 51^ underlining the validity of this approach. Second, we obtained neutrophils from CF adults with established lung disease and therefore established lung inflammation. An effect of inflammation on neutrophil death pathways in CF can therefore not be completely excluded by our data, although our data would suggest the mechanism is inflammation independent (particularly when we consider the results from *CFTR*^-/-^ piglets). Further investigation of these processes in neutrophils from children with CF before and after the onset of significant inflammatory lung disease would help address this in the future. Third, although we suggest an association of CFTR deficiency with delayed apoptosis that is corrected by ivacaftor therapy in patients with the G551D mutation, further studies of CFTR potentiation and correction and its effects on neutrophil processes such as apoptosis and NETosis will be required. The advent of newer combination therapies to correct and potentiate the more common CF mutations such as F508del will make this possible in the future,^52^ allowing the study of both in vivo and in vitro correction of CFTR on neutrophil functions. Furthermore, we would suggest that the CFTR null pig may also be used to investigate neutrophil function and death related to CFTR deficiency, as an extension of our data in this paper and as recently studied in CF pig macrophages.^33^

In summary, we demonstrate that CF neutrophils have prolonged survival secondary to decreased apoptosis, and this is likely a primary defect in CF neutrophils. Increased NET formation by CF neutrophils is a consequence of a failure to engage in apoptosis and can be targeted by CDKi drugs. NETs are a powerful proinflammatory stimulus to macrophages, with CF-derived macrophages being hyper-responsive to NET stimulation. In conclusion, an intrinsic delay in neutrophil apoptosis enhances NET formation in CF and consequently inflammation, representing a novel target for the development of future anti-inflammatory therapies.
