# Supplementary material for: Delayed neutrophil apoptosis enhances NET formation in cystic fibrosis
Source: Thorax. 2017 Sep 15;73(2):134–44. doi: 10.1136/thoraxjnl-2017-210134 (PMC5771859; doi:10.1136/thoraxjnl-2017-210134)
Supplement: Supplementary data [file thoraxjnl-2017-210134supp001.pdf]

Table E1.

Age	Sex	Genotype	FEV_1 (Liters)	Colonising Organsim
27	F	F508del/3659del.C	2.52	B Cen
33	M	F508del/F508del	2.02	PA, StM
29	M	F508del/F508del	2.12	PA
33	M	F508del/F508del	2.85	PA, B Viet
48	M	F508del/9T-7T	2.48	SA
27	F	F508del/F508del	1.45	PA
32	M	F508del/F508del	2.4	PA,HI
39	F	F508del/F508del	1.96	PA
34	F	F508del/P67L	1.32	B Mult
18	M	F508del/1717-1G>A	UK	SA, Pan Sp, PA
24	F	F508del/Gln493X	1.31	UK
20	F	F508del/F508del	1.49	PA
33	M	F508del/R117H	4.2	SA
25	M	F508del/c.4096-7A>G	3.58	SA
19	F	F508del/F508del	2.17	PA
25	M	F508del/F508del	1.89	MRSA
20	M	F508del/G551D	3.16	PA, StM
27	M	F508del/E60X	2.68	UK
19	M	F508del/F508del	3.71	SA

Table E1. Patient characteristics of CF patients providing samples for neutrophil apoptosis studies in Figures 1 and 3 (D and F). PA= Pseudomonas aeruginosa, SA= Staphylococcus aureus, HI= Haemophilus Influenzae, BC= Burkholderia cenocepacia, BViet= Burkholderia vietnamiensis, BMult= Burkholderia multivorans, StM= Stenotrophomonas maltophilia, MRSA= Methicillin Resistant Staphylococcus aureus. Pan Sp= Pandorea Sputorum UK=Unknown.

Table E2.

Age	Sex	Genotype
23	F	G551D/F508del
25	F	G551D/F508del
23	F	G551D/F508del
30	F	G551D/F508del
27	F	G551D/F508del
22	F	G551D/3659delC
33	F	G551D/F508del
57	F	G551D/P67L
35	F	G551D/F508del
29	M	G551D/G551D
30	M	G551D/F508del
33	M	G551D/R117H

Table E2. Patient characteristics of subjects providing neutrophil samples before and following Ivacaftor therapy utilized in Figure 2.

Table E3.

Age	Sex	Genotype
21	F	F508del/F508del
40	F	F508del/F508del
33	F	F508del/F508del
42	F	F508del/F508del
26	M	F508del/F508del
27	M	F508del/F508del
33	M	F508del/F508del
36	M	F508del/F508del
23	F	G542X/G551D
53	F	F508del/R117H
27	M	F508del/V520F
24	F	F508del/F508del
43	F	F508del/F508del
24	F	F508del/F508del
23	F	F508del/F508del
27	M	F508del/F508del
21	F	F508del/1717-8G->A
25	M	F508del/F508del
18	M	F508del/F508del

Table E3. Patient characteristics of subjects providing neutrophil samples utilized in Figures 3 (A-C), 4, 5 and 6.
