# Supplementary figures and images for: Delayed neutrophil apoptosis enhances NET formation in cystic fibrosis
Source: Thorax. 2017 Sep 15;73(2):134–44. doi: 10.1136/thoraxjnl-2017-210134 (PMC5771859; doi:10.1136/thoraxjnl-2017-210134)

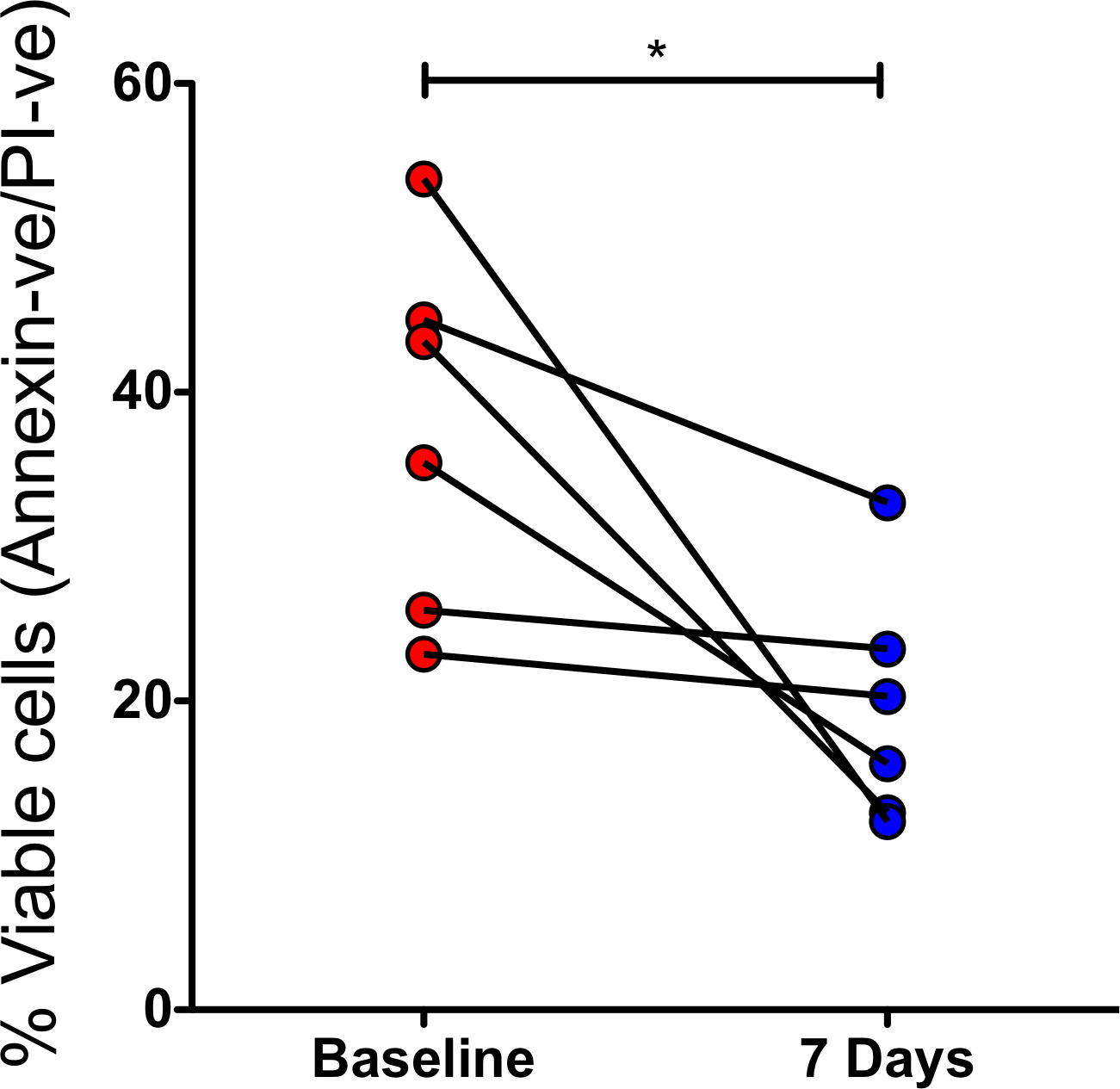

Supplement: Supplementary figure s1 [file thoraxjnl-2017-210134supp002.jpg]

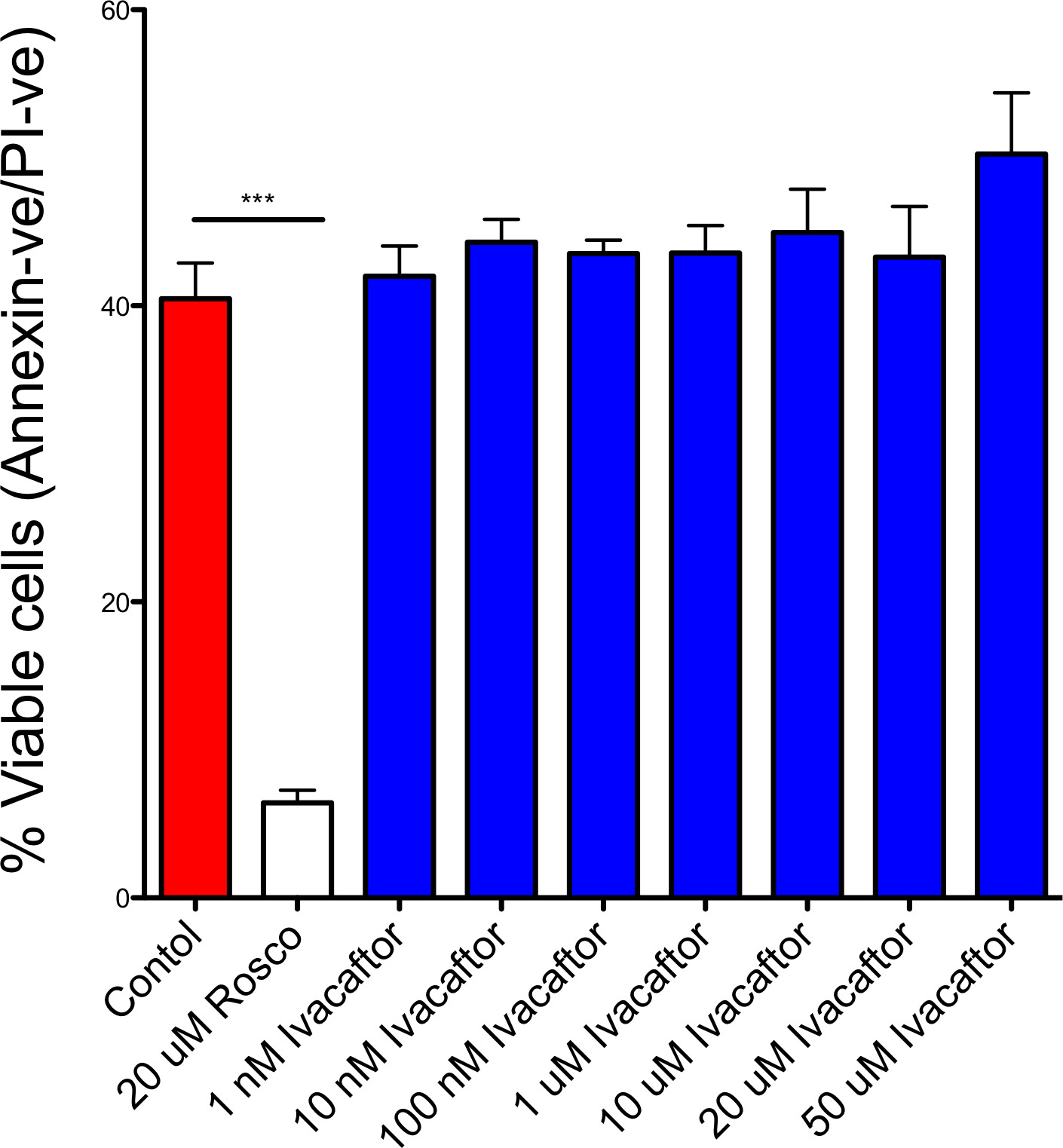

Supplement: Supplementary figure s2 [file thoraxjnl-2017-210134supp003.jpg]

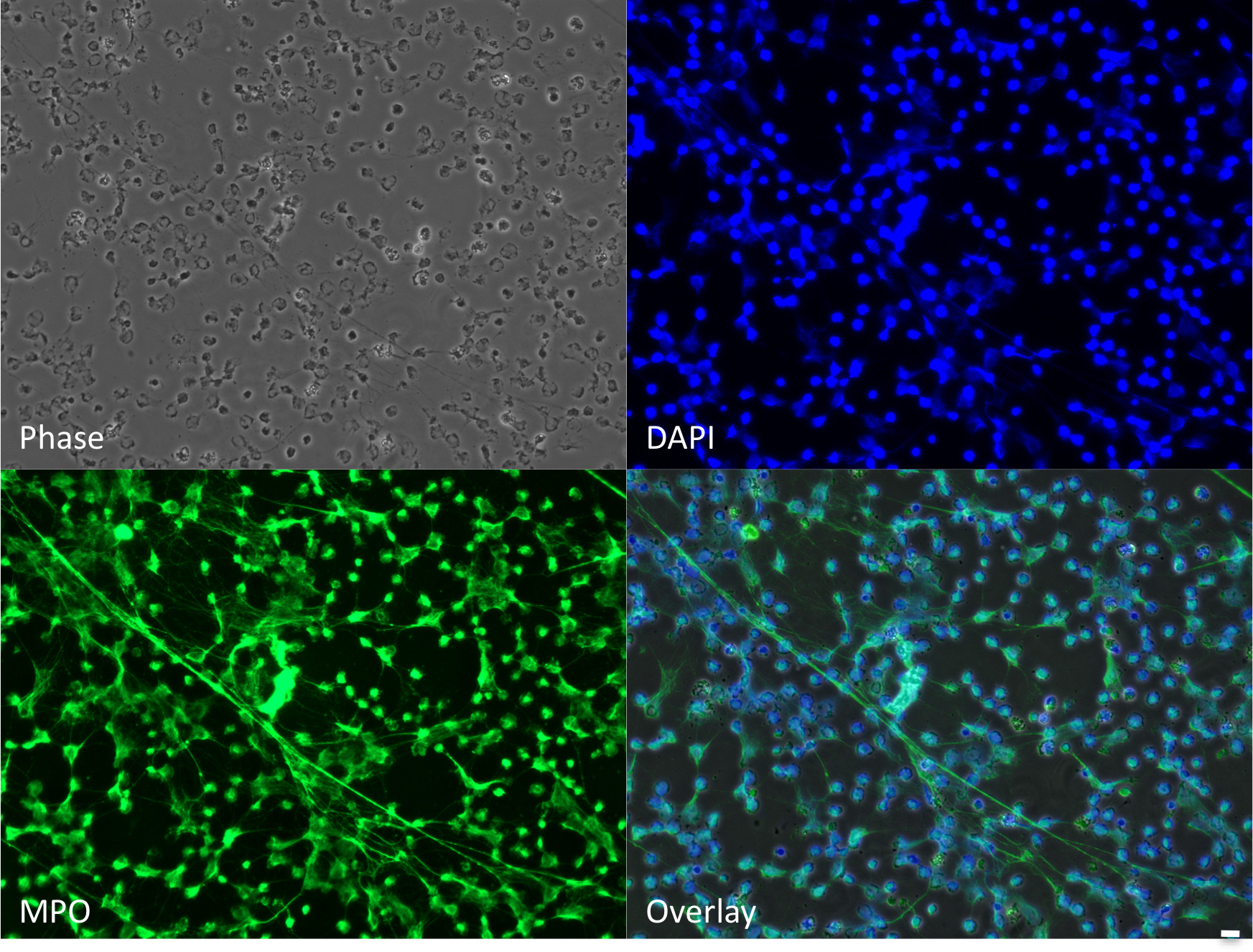

Supplement: Supplementary figure s3 [file thoraxjnl-2017-210134supp004.jpg]
